# Associations between obesity, weight change and decreased renal function in Korean type 2 diabetic patients: a longitudinal follow-up study

**DOI:** 10.1186/s12902-021-00853-z

**Published:** 2021-09-17

**Authors:** Bo-Yeon Kim, Dug-Hyun Choi, Chan-Hee Jung, Ji-Oh Mok, Chul-Hee Kim

**Affiliations:** Division of Endocrinology and Metabolism, Department of Internal Medicine, Soonchunhyang University Bucheon Hospital, Soonchunhyang University College of Medicine, 170 Jomaru-ro, Wonmi-gu, Bucheon, 14584 Republic of Korea

**Keywords:** Obesity, Body weight changes, Diabetes mellitus, Type 2, Decreased renal function

## Abstract

**Background:**

We aimed to examine the associations between the risk of decreased renal function, obesity, and weight changes in Korean type 2 diabetic patients with normal renal function.

**Methods:**

Type 2 diabetic patients (*n* = 1060) who visited the diabetic clinic at Soonchunhyang University Bucheon Hospital between 2001 and 2007 with follow up surveys completed in 2016 to 2017 were recruited into the study. Decreased renal function was defined as an estimated glomerular filtration rate < 60 mL/min/1.73 m^2^. Weight change was calculated between baseline and each follow-up survey. Multivariate analysis was used to evaluate the longitudinal association of baseline obesity and weight changes with the risk of decreased renal function.

**Results:**

This study revealed that baseline obesity was associated with the risk of decreased renal function after adjusting for clinical variables in type 2 diabetic patients (odds ratio [OR] 1.40; 95% confidence intervals [CI] 1.08–2.04; *p* = 0.025). Follow-up (mean = 12 years) revealed that weight gain > 10% was associated with the risk of decreased renal function after adjusting for clinical variables in type 2 diabetic patients with normal renal function at baseline (OR 1.43; CI 1.11–2.00; *p* = 0.016). Weight loss was not associated with the risk of decreased renal function in type 2 diabetic patients with normal renal function at baseline.

**Conclusions:**

Baseline obesity was associated with the increased risk of decreased renal function in Korean type 2 diabetic patients with normal renal function. Weight gain > 10% independently predicted the risk of decreased renal function. Large prospective studies are needed to clarify causal associations between obesity, weight change, and decreased renal function in patients with type 2 diabetes.

## Background

Obesity is a major risk factor for type 2 diabetes, hypertension, and other comorbidities [1]. In Korea, the prevalence of diabetes has increased due to an aging population [2]. In 2016, diabetes was the most common cause of renal replacement therapy in Korea [3]. The prevalence of chronic kidney disease (CKD) among adults was reported as 8.2% in a nationwide representative sample of the Korean population [4]. Clinical factors such as aging, diabetes, and hypertension may be associated with the increasing prevalence of CKD [4, 5]. Recently, a link between obesity and decreased renal function has been identified. Obesity has been reported to promote renal dysfunction by several mechanisms including alterations in renal hemodynamics and inflammation [6, 7]. A meta-analysis suggested that obesity has a U-shaped association with the risk of kidney disease in patients with hypertension [8]. Conversely, some studies reported that high body mass index (BMI) is protective for renal function deterioration in type 2 diabetes and CKD [9, 10].

Since the prevalence of obesity in diabetic patients is increasing [11], and diabetes is the main cause of CKD [5], it is important to understand how changes in body weight and waist circumference affect the progression of CKD in patients with diabetes and chronic disease. To our knowledge, few studies have examined the effects of weight change on the risk of developing CKD in Asian patients with type 2 diabetes. In present study, we investigated the associations between the risk of decreased renal function, obesity, and weight changes in Korean type 2 diabetic patients with normal renal function.

## Methods

### Subjects

Korean patients with type 2 diabetes (*n* = 1060) who visited the diabetes clinic at Soonchunhynag University Bucheon Hospital between 2001 and 2007 with follow-up surveys completed in 2015 to 2017 were recruited into the study. Individuals diagnosed with type 1 diabetes, renal insufficiency (estimated glomerular filtration rate [eGFR] < 60 mL/min/1.73 m^2^), chronic liver disease, chronic infection, or malignancy were excluded. Decreased renal function was defined as an eGFR < 60 mL/min/1.73 m^2^. Based upon the Modification of Diet in Renal Disease formula, eGFR was calculated as follows: eGFR (mL/min/1.73 m2) = 186 × serum creatinine (mg/dL) - 1.154 × (age) - 0.203 × (0.742 if female).

BMI was categorized as normal (< 23 kg/m^2^), overweight (23–24.9 kg/m^2^), or obese (≥25 kg/m^2^) in accordance with WHO guidelines for the Asia-Pacific region [12] and 2018 Korean Society for the Study of Obesity Guideline [13]. Weight change was calculated between baseline measurements and that at the time of each follow-up survey. Relative risk of decreased renal function and 95% confidence intervals (CI) were estimated. To estimate the risk of decreased renal function, associations were examined in patients who were followed up regularly (*n* = 861). The study protocol conformed to the ethical guidelines of the 1975 Declaration of Helsinki and was approved by the Institutional Review Board (IRB) of Soonchunhyang University Bucheon Hospital (IRB no. 2020–03-015).

### Measurements

At the initial visit, fasting glucose, glycated hemoglobin (HbA1c), fasting total serum cholesterol, triglyceride, high-density lipoprotein cholesterol, low-density lipoprotein cholesterol, aspartate aminotransaminase, alanine aminotransferase (ALT), and creatinine were measured. Serum creatinine was assayed by calorimetry using Beckman Coulter AU analyzer (Beckman Coulter Inc., Brea, CA, USA) following the Jaffe method and it was IDMS standardized [14]. HbA1c was measured using ion-exchange high-performance liquid chromatography (Bio-Rad, Hercules, CA, USA) (4.0–6.0%). Albuminuria was measured using a radioimmunoassay (Immunotech, Marseille, France) using spot urine or time-collected urine. Microalbuminuria was defined as an albumin excretion rate of 20–200 μg/min, an albumin/creatinine ratio in spot urine of 30–300 mg/g, or a 24-h urine protein of 30–300 mg/day. Overt albuminuria was defined as an albumin excretion rate > 200 μg/min, an albumin/creatinine ratio in spot urine of > 300 mg/g, or a 24-h urine protein of > 300 mg/day. All laboratory tests were measured in one laboratory.

### Statistical analysis

Statistical analyses were performed using SPSS Statistics version 26.0 (IBM Corp., Armonk, NY, USA). Values are represented as mean ± standard deviation for variables that were normally distributed, median (interquartile range) for variables that were not normally distributed, or the number of participants (percentages). *P*-values less than 0.05 were considered to be statistically significant. A chi-square test was performed to compare categorical variables. One-way analysis of variance was performed to evaluate the differences in means among BMI categories. Multivariate logistic regression was performed to identify the longitudinal associations of baseline obesity and weight changes with the risk of decreased renal function.

## Results

The baseline characteristics of study participants are presented in Table 1. Subjects (*n* = 1060) were analyzed and divided into three groups according to BMI categories as described in Materials and Methods. The prevalence of obesity (BMI ≥25 kg/m^2^) was 45.8% in subjects with type 2 diabetes. Obese patients had higher HbA1c, higher ALT, and a less favorable lipid profile, whereas no differences were found between the three BMI groups in eGFR or the prevalence of albuminuria (Table 1).

**Table 1:** Baseline characteristics of 1060 participants with type 2 diabetes according to the body mass index categories

Characteristic	BMI < 23 (***n*** = 320, 30.2%)	23 ≤ BMI < 25 (***n*** = 255, 24.1%)	BMI ≥ 25 (***n*** = 485, 45.8%)	***p***
Age (year)	56.3 ± 8.4	56.7 ± 8.5	54.1 ± 8.7	0.002
Sex (%)				0.003
Men	44.6	53.0	42.3	
Women	55.4	47.0	57.7	
BMI (kg/m^2^)	21.5 ± 1.3	24.1 ± 1.3	29.4 ± 2.7	< 0.001
Duration of type 2 diabetes (year)	6.5 ± 5.3	5.0 ± 4.5	5.5 ± 5.4	NS
HbA1c (%)	7.4 ± 1.5	7.3 ± 1.7	8.3 ± 1.7	0.005
Fasting glucose (mg/dL)	133.6 ± 33.1	175.1 ± 57.3	180.3 ± 47.3	< 0.001
AST (U/L)	35.6 ± 9.6	36.3 ± 11.2	36.8 ± 10.3	NS
ALT (U/L)	39.6 ± 10.4	41.9 ± 14.7	45.8 ± 1.7	0.002
Total-cholesterol (mg/dL)	160.9 ± 32.5	186.2 ± 43.8	192.0 ± 48.7	0.002
LDL-cholesterol (mg/dL)	93.3 ± 27.4	102.3 ± 40.7	106 ± 50.7	0.001
HDL-cholesterol (mg/dL)	43.3 ± 10.6	46.5 ± 16.1	42.5 ± 16.1	NS
Triglycerides (mg/dL)	155.7 ± 67.6	177.9 ± 131.3	197.9 ± 141.3	0.001
Hypertension (%)	61.5	68.0	71.0	0.040
eGFR (mL/min/1.73 m^2^)^a^	75.2 ± 11.1	74.6 ± 13.9	70.5 ± 13.0	0.002
Albuminuria (%)	31.6	29.0	30.5	NS

The data is presented as mean ± standard deviation

*BMI* body mass index, *HbA1c* glycated hemoglobin, *AST* aspartate aminotransaminase, *ALT* alanine aminotransferase, *LDL* low-density lipoprotein, *HDL* high-density lipoprotein, *eGFR* estimated glomerular filtration rate, *NS* not significant

^a^Calculation of eGFR based upon the Modification of Diet in Renal Disease formula: eGFR (mL/min/1.73 m^2^) = 186 × serum creatinine (mg/dL) - 1.154 × (age) - 0.203 × (0.742 if female) × (1.21 if black)

**Fig. 1:**
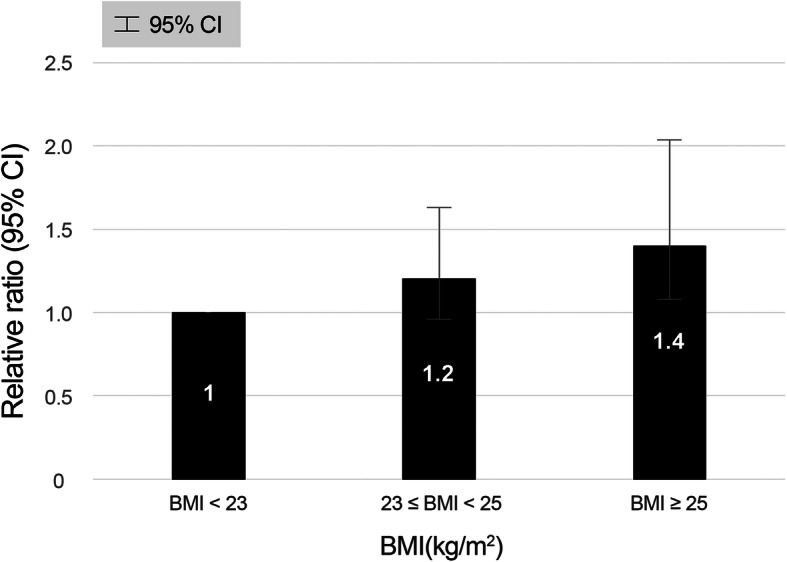
Relative risk of decreased renal function according to baseline body mass index categories adjusted for age, sex, duration of diabetes, glycated hemoglobin, total cholesterol, triglycerides, and hypertension (high blood pressure or anti-hypertensive medication use). *BMI,* body mass index; *CI,* confidence interval

Baseline obesity was associated with higher odds ratios (OR) for decreased renal function after adjusting for clinical factors such as age, sex, duration of type 2 diabetes, HbA1c, total cholesterol, triglycerides, and hypertension at baseline (OR 1.40; CI 1.08–2.04; *p* = 0.025) (Table 2, Fig. 1). The overweight group did not have a higher risk of decreased renal function (OR 1.20; CI 0.96–1.63; *p* = 0.102) (Table 2).

**Table 2:** Longitudinal association between baseline obesity and risk of decreased renal function (estimated glomerular filtration rate < 60 mL/min/1.73 m^2^) (*n* = 1060)

	Age-adjusted		Model 1^a^		Model 2^b^	
	RR (95% CI)	***p***	RR (95% CI)	***p***	RR (95% CI)	***p***
BMI (kg/m^2^)						
BMI < 23	1 (reference)		1 (reference)		1 (reference)	
23 ≤ BMI < 25	1.11 (0.93–1.59)	0.190	1.24 (0.96–1.65)	0.090	1.20 (0.96–1.63)	0.102
BMI ≥ 25	1.36 (1.07–2.00)	0.020	1.44 (1.12–2.12)	0.017	1.40 (1.08–2.04)	0.025

*BMI* body mass index, *RR* relative risk, *CI* confidence interval

^a^Model 1: adjusted for age, sex, and duration of type 2 diabetes

^b^Model 2: adjusted for Model 1 + glycated hemoglobin, total cholesterol, triglycerides, and hypertension (high blood pressure or anti-hypertensive medication use)

**Fig. 2:**
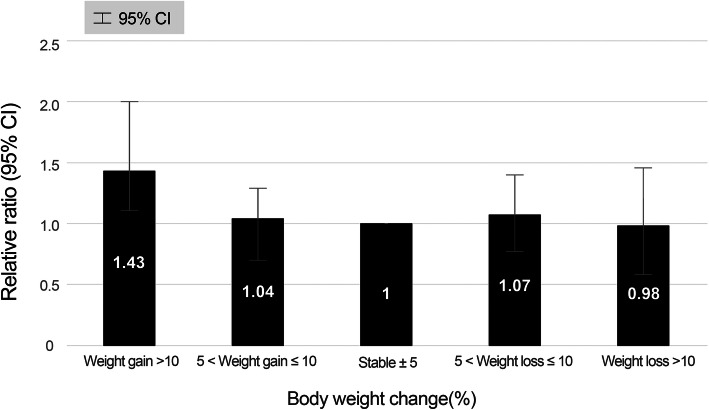
Relative risk of decreased renal function according to body weight changes categories adjusted for age, sex, duration of diabetes, glycated hemoglobin, total cholesterol, triglycerides, and hypertension (high blood pressure or anti-hypertensive medication use). *CI,* confidence interval

In type 2 diabetic patients with normal renal function, weight gain > 10% was associated with the risk of decreased renal function during follow-up (mean = 12 years) after adjusting for clinical variables at baseline (OR 1.43; CI 1.11–2.00; *p* = 0.016). However, weight gain of 5–10% and weight loss of 5–10% were not associated with the risk of decreased renal function in type 2 diabetic patients with normal renal function at baseline. Moreover, weight loss of any magnitude was not associated with the risk of decreased renal function in type 2 diabetic patients with normal renal function (OR 0.98; CI 0.58–1.46; *p* = 0.719) (Table 3, Fig. 2).

**Table 3:** Longitudinal association of body weight changes and risk of decreased renal function (estimated glomerular filtration rate < 60 mL/min/1.73 m^2^) (*n* = 861)

	Age-adjusted		Model 1^a^		Model 2^b^	
	RR (95% CI)	***p***	RR (95% CI)	***p***	RR (95% CI)	***p***
Body weight change (%)						
Weight gain > 10	1.50 (1.12–2.01)	0.003	1.44 (1.11–2.00)	0.005	1.43 (1.11–2.00)	0.016
5 < weight gain ≤10	1.03 (0.70–1.29)	0.710	1.03 (0.70–1.29)	0.740	1.04 (0.70–1.29)	0.634
Stable ±5	1 (reference)		1 (reference)		1 (reference)	
5 < weight loss ≤10	1.07 (0.77–1.40)	0.450	1.05 (0.77–1.40)	0.457	1.07 (0.77–1.40)	0.519
Weight loss > 10	0.98 (0.58–1.46)	0.602	0.99 (0.58–1.48)	0.702	0.98 (0.58–1.46)	0.719

*RR* relative risk, *CI* confidence interval

^a^Model 1: adjusted for age, sex, and duration of type 2 diabetes

^b^Model 2: adjusted for Model 1 + glycated hemoglobin, total cholesterol, triglycerides, and hypertension (high blood pressure or anti-hypertensive medication use)

## Discussion

Our study demonstrates that baseline obesity is associated with the risk of decreased renal function and that weight gain > 10% is an independent predictor of decreased renal function in Korean type 2 diabetic patients with normal renal function. Overweight patients (23 ≤ BMI < 25) did not have a higher risk of decreased renal function. Weight loss and weight gain < 10% were not associated with the risk of decreased renal function in type 2 diabetic patients with normal renal function at baseline.

In several population-based studies, higher BMI was associated with the presence of CKD [15, 16], decreased eGFR [17–19], and incidental end-stage renal disease [20, 21]. Our findings in Korean patients with type 2 diabetes are consistent with the results of previous studies. It is thought that because type 2 diabetes is a major cause of CKD and those with type 2 diabetes represent a population with different health characteristics than healthy subjects, the specific study of CKD in diabetic patients is of importance. Higher BMI was reported as an independent predictor of major renal events in patients with type 2 diabetes [22]. In Asian diabetics, obesity was shown to be associated with increased albuminuria and diabetic kidney disease [23]. However, some studies of Asian diabetic patients have demonstrated a negative correlation between obesity and new onset of CKD (eGFR < 60 mL/min/1.73 m^2^) [9, 24].. According to the Hong Kong Diabetes Registry, CKD is positively correlated with central obesity and negatively correlated with BMI [9]. Sarcopenia due to malnutrition is common in CKD patients, resulting in a decrease in BMI with subsequent effects on clinical outcomes. A previous study described this observation as a reverse causality [25]. BMI may not be a reliable marker of outcome in CKD patients [26]. Extensive research is needed to clarify these controversies.

Patients with type 2 diabetes have several comorbidities [11]. It is important to take into consideration that weight gain can affect hypertension, dyslipidemia, and hyperglycemia. The understanding of the effects of weight gain on the development of CKD is important, which was the objective of the present study encompassing these clinical factors.

In this study, baseline body weight was associated with a higher risk of incidental CKD. Several recent studies demonstrated the association between obesity and increased risk of CKD. However, few studies have investigated whether weight gain increases the risk for CKD. In a Japanese study, the relative risk for incident CKD was higher in the normal weight participants with a weight gain of > 10 kg than those without weight gain [27].

In another study in Europeans, waist circumference (WC) changes were not associated with the incident CKD in women, whereas a severe increase in WC was associated with increased risk of CKD [28]. The effect of obesity on CKD may be different between men and women.

Factors involved in the progression of CKD and mechanisms by which obesity may affect CKD are not fully clarified yet. However, several mechanisms have been proposed. Various metabolic diseases associated with obesity may lead to kidney damage. The association between metabolic syndrome and kidney disease is well known [29, 30]. Obesity has a direct effect on the kidneys. Adiposity leads to the dysfunction of adipocytokines, such as adiponectin [31], leptin [32], and resistin [33], which may affect the kidneys. Increased inflammation, oxidative stress [34], increased renin-angiotensin-aldosterone system [35], and insulin resistance [36] are known as mechanisms of renal failure in obesity. Specific pathologic changes associated with obesity such as hyperfiltration-related glomerulopathy [37] and increased nephrolithiasis [38] may be important CKD-related mechanisms. In a study of type 2 diabetes, an increase in IL-6 associated with weight gain was shown to be related to an increased risk of CKD [39].

In the present study, weight loss was not associated with decreased renal function. Similar to our findings, a decrease in WC was not related to a lower risk of CKD in another recent study [28]. In another study, intensive lifestyle intervention concentrating on weight loss could not decrease cardiovascular events in overweight or obese patients with type 2 diabetes but delayed the rate of progression [40]. In type 2 diabetic patients with various metabolic diseases, weight loss may hinder the progression of various metabolic diseases thereby preventing CKD and protecting the kidneys [40].

This study has several limitations. First, we focused on GFR in this study. Obesity is an important cause of proteinuria, such as obesity related nephropathy. Further study is needed to observe the association between obesity and proteinuria during the long-term follow-up. Second, the single-center design may introduce selection bias. However, the laboratory results of this study population are highly consistent with the broader Asian population. Despite these limitations, our study has the advantage of a long mean follow up of 12 years.

In conclusion, obesity was associated with the increased risk of decreased renal function in Korean type 2 diabetic patients with normal renal function. Weight gain > 10% independently predicted decreased renal function. Large prospective studies are needed to clarify causal associations between obesity, weight change, and decreased renal function in patients with type 2 diabetes.

## Data Availability

Restrictions apply to the availability of these data, which were used under license for the current study, and so are not publicly available. Administrative permissions from the IRB of the Soonchunhyang University Bucheon Hospital are required to access the raw data.

## Abbreviations

ALT: Alanine aminotransferase
AST: Aspartate aminotransaminase
BMI: Body mass index
CI: Confidence intervals
CKD: Chronic kidney disease
eGFR: Estimated glomerular filtration rate
HbA1c: Glycated hemoglobin
HDL: High-density lipoprotein
IRB: Institutional Review Board
LDL: Low-density lipoprotein
NS: Not significant
OR: Odds ratio
RR: Relative risk
T2DM: Type 2 diabetes mellitus
WC: Waist circumference
